# Looking back at paediatric HIV treatment in South Africa. My, how we have grown!

**DOI:** 10.4102/sajhivmed.v22i1.1283

**Published:** 2021-09-16

**Authors:** Leon J. Levin, Juliet L. Horak, James Nuttall

**Affiliations:** 1 Department of Paediatrics, Right to Care, Johannesburg, South Africa; 2 Department of Paediatrics, Dora Nginza Hospital, Gqeberha, South Africa; 3 Department of Paediatrics, Faculty of Health Sciences, University of Cape Town, Cape Town, South Africa

**Keywords:** HIV, paediatric HIV, paediatric antiretroviral treatment, South Africa, history

## Abstract

Antiretroviral treatment has undergone major changes in the last 20 years, from monotherapy, to dual therapy and finally to triple therapy. Lately, more focus has been placed on better, more well-tolerated combinations and formulations. As in most other disciplines in medicine, the development of paediatric HIV dosages and formulations always tends to lag behind adult research. Twenty years ago, it could take several years before data were available to enable the use of life-saving antiretrovirals in children. Paediatricians, being ever resourceful, were not prepared to let their paediatric patients suffer despite the lack of data or formulations and so made a plan. This article describes some of the trials and tribulations that we went through trying to make sure that our paediatric HIV patients not only survived but thrived. Clinicians treating paediatric patients today have it so much easier because of what our colleagues and their patients went through in those early days.

It is hard to convey the sense of helplessness felt by those of us who worked in the pre-antiretroviral treatment (ART) era when faced with a sick HIV-infected child and mother. We had little to offer except the treatment of opportunistic infections and co-trimoxazole prophylaxis. We have come a long way since then.

In the mid-1990s, when HIV treatment had evolved only as far as dual nucleoside reverse transcriptase inhibitors (NRTIs), an article appeared in the *New England Journal of Medicine* suggesting that didanosine (ddI) monotherapy was as good as the combination of zidovudine (AZT) and ddI and better than AZT alone.^1^ At the time, viral load monitoring was unavailable, and the less useful marker of HIV disease progression and/or death was used in clinical trials. Because ddI monotherapy was quite affordable (R80.00 a month for a baby), a few patients in the state sector were able to afford ddI monotherapy, and this did provide a brief respite from the ravages of the disease.

In 1998, two developments spurred the cause of paediatric HIV treatment in South Africa. The first was the launch of stavudine (d4T) in South Africa. We now know that this is a very toxic drug, but in those days, it was heralded as a new, very potent NRTI. However, no matter how low the required paediatric dosage, d4T remained expensive and often unaffordable, at approximately R1000.00 a month. To help our patients, one of us would purchase a month’s supply of the adult 40 mg capsules costing R1000.00 and have a chemist split the contents of the capsule into two and put each half back into another capsule. That way, the 20 mg capsules were made available at half price. Stavudine powder for reconstitution with water to form a suspension of 1 mg/mL and dosed at 1 mg/kg per dose twice a day was subsequently released but required refrigeration and the administration of relatively large volumes of liquid medication to young children. To get around these problems, many clinicians treating children unable to swallow capsules or in whom a lower dose was required used a technique of opening d4T capsules, dispersing the powder contents in water and administering an appropriate weight-based dose. Some years later, pharmacological and pharmacokinetic evaluations using high-performance liquid chromatography confirmed the accuracy of the off-label opened-capsule dosing method for stavudine and showed that plasma drug exposure after stavudine administration as a solution in this way was bioequivalent to intact capsule administration.^2,3^

However, ARVs were still very expensive, and few individuals could afford them. They were not available in the state sector, and medical aids were generally not paying for them, either. In 1998, Aid for AIDS (AfA) was launched to assist medical insurance schemes to manage HIV in the private sector. It was revolutionary, but there was a limit – adult patients could only receive dual therapy and not triple therapy. One of the researchers (L.J.L.) approached AfA (Dr Leon Regensburg) and asked if it would be possible for paediatric patients to receive triple therapy, because they could get three drugs for the same price as two drugs in adults. AfA did, as a result, allow paediatric patients triple therapy; this is why some paediatric patients in the private sector received triple therapy as early as 1998. Although many were on d4T/ddI/ritonavir (RTV) – not a great regimen in anybody’s book – many survived and are alive and well today. While paediatric ARV experience was evolving in the private sector, nothing was available in the state sector. Hydroxyurea, a cheap drug used in oncology and believed to be synergistic with ddI, was tried. Because of its low cost, patients, including children, were treated with the combination.^4,5^ However, its toxicity – lactic acidosis, often with fatal consequences – was quickly recognised, and this therapy ended.

From 2000, things changed for the better. The AIDS 2000 conference was a watershed moment, as was the launch of the *Southern African Journal of HIV Medicine*. In the early 2000s, very limited access to paediatric ART in the public sector was mostly via international humanitarian non-governmental organisations such as Médecins sans frontiers (Doctors Without Borders) and donor-supported hospital-based programmes.^6,7^ On 01 April 2004, the national ARV roll-out began.^8^ This was wonderful, but paediatric formulations were far from optimal. Children were given d4T/lamivudine (3TC) combined with lopinavir/r (LPV/r) if they were under 3 years of age or efavirenz if they were over 3 years. Besides the problem of the terrible taste and poor tolerability of LPV/r, it was not used in children under 6 months in the absence of data on its use in this age group. So, any child under 6 months was given full-dose RTV, as were any patients on concurrent rifampicin-containing antituberculosis treatment. Besides the bad taste of RTV, this protease inhibitor (PI) presents HIV with a low genetic barrier to resistance, resulting in many children developing PI resistance.^9^ Several years later, the NRTI backbone was changed to abacavir and 3TC, a safer combination and one that allowed for once-daily dosing – a big help in terms of adherence.

One of the obstacles to achieving widespread coverage of paediatric ART has been the simplification of the prescribing process for non-paediatricians, including doctors and nurses who are more familiar with prescribing ART for adults but who are also involved in treating children, particularly at the primary care level. Calculating individualised doses of separate and mostly liquid oral ARV formulations for an infant or young child at each clinic visit using the current weight or body surface area is complicated and time-consuming. The development and updating of an integrated weight-based ARV dosing chart for children based on World Health Organization guidelines and adapted for the ARV formulations available in South Africa has contributed to building confidence amongst prescribing clinicians and pharmacists and helped facilitate children’s access to ART.

However, issues of ARV tolerability and access to formulations appropriate for children remain. The LPV/r formulation is very unpleasant to taste, and whilst young babies tend to tolerate it when their taste buds are still undeveloped, as they grow older, they often spit or vomit it out. Only in 2020 did the LPV/r pellets become available both in the state and private sectors. However, in the state sector, it may only be prescribed for patients over 6 months of age who are not tolerating the LPV/r solution.^10^ In the 2019 national guidelines, tenofovir/3TC/dolutegravir fixed-dose-combination tablets can be used from 10 years of age and 35 kg upwards,^11^ making a well-tolerated single-tablet regimen available for the first time to these adolescents. Another issue that is still a problem is adolescent disclosure, because very few healthcare workers feel comfortable disclosing to their adolescent patients, and parents are obviously petrified to do so because of feelings of guilt and worries that the children may be angry with them. This and other adolescent issues are resulting in very low rates of adolescent viral suppression.^12^

In South Africa, HIV viral resistance testing first became available in the early 2000s. At the time, a prevailing practice was to keep children with high viral loads on the same ART regimen as long as the CD4 count was acceptable. This was a pragmatic approach because of the limited treatment options available and the restricted access to resistance testing but is likely to have contributed to the accumulation of progressive resistance mutations in some children, thereby limiting future treatment options. A national third-line ART expert committee was established in 2013, focusing on patients with virological failure on a PI-based ART regimen. New-generation PIs and the integrase strand transfer inhibitor (INSTI) class of ARV drugs became accessible, though on a limited basis. This has provided real hope for treatment-experienced adults and children and their clinicians in achieving better long-term treatment outcomes.

Alongside the changes in paediatric ART, the reduction in mother-to-child transmission (MTCT) resulting from maternal ART has been dramatic. Between 2009 and 2015, new paediatric infections decreased by 84% as a result of our national prevention of MTCT guidelines evolving rapidly,^13^ culminating in January 2015 in the provision of lifelong triple ART for all pregnant and breastfeeding women. Whilst fewer children are acquiring HIV through vertical transmission, we are finding these children particularly challenging to deal with. Pregnant women who know their HIV status and are able to take ART rarely transmit HIV to their babies. Of course, some mothers acquire HIV for the first time during their pregnancy or whilst breastfeeding and have unfortunately already transmitted the infection to their baby before they are aware of their status. However, the majority of infected babies in the era of ART are born to mothers who for complex social reasons have defaulted on their ART or take it inconsistently. Poverty, stigma, and drug and alcohol abuse are all contributory factors. Unfortunately, many of these mothers in turn find it difficult to give ARVs to their children and to access treatment for them. Many of the children who acquire HIV now are born into families with the least ability to deal with it. The hopelessness we felt in the pre-ART era is now replaced by the heartbreak and frustration of having life-saving treatment that is not taken.

In summary, paediatric ART in South Africa has come a long way: monotherapy, dual therapy, toxic regimens with d4T, ddI, and RTV. However, a universally available, well-tolerated, single formulation available from birth to adolescence still eludes us. Whilst advocating for new, simpler and better-tolerated paediatric regimens, an underlying constraint remains – that of improving the support of vulnerable patients, especially pregnant and breastfeeding mothers, so that they are able to take ART consistently and ensure the protection of the next generation of South Africans.

## References

[1] Englund JA, Baker CJ, Raskino C, et al. Zidovudine, didanosine, or both as the initial treatment for symptomatic HIV-infected children. N Engl J Med. 1997;336:1704–1712. 10.1056/NEJM199706123362403 9182213

[2] Innes S, Smuts M, Cotton M, Seifart H, Rosenkranz B. Comparative study of different brands of stavudine capsules for the off-label ‘opened capsule’ dosing method recommended for HIV-infected infants and children in resource-limited settings. SAJCH. 2009 7;3(2):44–47. 20640190 PMC2905170

[3] Innes S, Norman J, Smith P, et al. Bioequivalence of dispersed stavudine: Opened versus closed capsule dosing. Antivir Ther. 2011;16(7):1131–1134. 10.3851/IMP1876 22024529 PMC5054241

[4] Zachary KC, Davis B. Hydroxyurea for HIV infection. AIDS Clin Care. 1998 4;10(4):25–26, 32. 11365149

[5] Sanne I, Smego RA. Systematic review of combination antiretroviral therapy with didanosine plus hydroxyurea: A partial solution to Africa’s HIV/AIDS problem? Int J Infect Dis. 2001;5:43–48. 11285159 10.1016/s1201-9712(01)90048-7

[6] Reddi A, Leeper S, Grobler A, et al. Preliminary outcomes of a paediatric highly active antiretroviral therapy cohort from KwaZulu-Natal, South Africa. BMC Pediatr. 2007;7:13. 10.1186/1471-2431-7-13 17367540 PMC1847430

[7] Eley B, Nuttall J, Davies M-A, et al. Initial experience of a public sector antiretroviral treatment programme for HIV-infected children and their infected parents. S Afr Med J. 2004;94(8):643–646. 15352588

[8] Simelela NP, Venter WDF. A brief history of South Africa’s response to AIDS. S Afr Med J. 2014;104(3 Suppl 1):249–251. 24893502 10.7196/samj.7700

[9] Van Zyl GU, Van der Merwe L, Claassen M, et al. Protease inhibitor resistance in South African children with virologic failure. Pediatr Infect Dis J. 2009 12;28(12):1125–1127. 19779394 10.1097/INF.0b013e3181af829d

[10] National Department of Health Circular. Notice: Availability of Lopinavir/ritonavir oral pellets to children on antiretroviral treatment, version 2021 [homepage on the Internet]. Reference 2021/22/02/EDP/02. [cited 2021 Jun 22]. Available from: https://drive.google.com/file/d/1uFlscKm0IwGEeUhxjkecCL9HfPzNEsjj/view?usp=sharing

[11] South African National Department of Health. National Consolidated Guidelines for the Management of HIV in adults, adolescents, children and infants and prevention of mother-to-child transmission [homepage on the Internet]. 2020 [cited 2021 Jun 22]. Available from: https://www.knowledgehub.org.za/system/files/elibdownloads/2020-07/National%20Consolidated%20Guidelines%2030062020%20signed%20PRINT%20v7.pdf

[12] Nglazi M, Kranzer K, Holele P, et al. Treatment outcomes in HIV-infected adolescents attending a community-based antiretroviral therapy clinic in South Africa. BMC Infect Dis. 2012;12:21. 10.1186/1471-2334-12-21 22273267 PMC3295677

[13] Joint United Nations Programme on HIV/AIDS (UNAIDS). On the fast-track to an AIDS-free generation. The incredible journey of the global plan towards the elimination of new HIV infections among children by 2015 and keeping their mothers alive. Geneva: Joint United Nations Programme on HIV/AIDS; 2016.

